# Concurrent bronchopulmonary foregut malformations: a rare case of right-sided extralobar pulmonary sequestration and bronchogenic cyst

**DOI:** 10.1186/s43055-021-00440-1

**Published:** 2021-02-16

**Authors:** Carolyn Hanna, Priya G. Sharma, Moiz M. Mustafa, Jennifer Reppucci, Archana Shenoy, Dhanashree Rajderkar

**Affiliations:** 1 Department of Radiology, University of Florida College of Medicine, 1600 SW Archer RD, Gainesville, FL 32608 USA; 2 Department of Pediatric Surgery, University of Florida College of Medicine, 1600 SW Archer RD, Gainesville, FL 32608 USA; 3 Department of Pathology, University of Florida College of Medicine, 1600 SW Archer RD, Gainesville, FL 32608 USA

**Keywords:** Bronchopulmonary foregut malformation, Pulmonary sequestration, CPAM, Bronchogenic cyst, Foregut duplication cyst

## Abstract

**Background:**

Bronchopulmonary foregut malformations are rare congenital malformations. It is extremely rare to have malformations that occur simultaneously. There is literature to show that extralobar sequestration is associated with other congenital anomalies, most commonly diaphragmatic hernias, and also with other bronchopulmonary foregut malformations (e.g., extralobar sequestration and congenital pulmonary airway malformations). However, very few case reports were found that reported extralobar sequestration and foregut duplication cysts and only one report of a right-sided complex foregut malformation with pulmonary sequestration.

**Case presentation:**

We present a case of a 3-month-old male infant with a prenatal diagnosis of a cystic lung lesion who, after developing symptoms of respiratory distress, was found to have concurrent right-sided extralobar pulmonary sequestration and a mediastinal bronchogenic cyst.

**Conclusions:**

The concurrent occurrence of these malformations in one patient could help support the theory that these malformations result from an early error in development during the time when both the lung buds and foregut are developing simultaneously.

## Background

Bronchopulmonary foregut malformations are rare congenital malformations resulting from anomalous budding of the tracheobronchial tree and primitive foregut. These lesions can be diagnosed in the antenatal period using prenatal ultrasound. Postnatal presentation is varied and can include cough, respiratory distress, and difficulty with feeding [1]. Surgical resection is the preferred method of treatment. The concurrent occurrence of multiple malformations has been documented, with several cases demonstrating an association between extralobar pulmonary sequestration and foregut duplication cysts. In this report, we present a unique case of a 3-month-old male infant who was prenatally diagnosed with a cystic lung lesion and developed respiratory distress postnatally with the discovery of right-sided extralobar sequestration and concurrent bronchogenic cyst.

## Case presentation

A 3-month-old male infant with a prenatal ultrasound diagnosis of a right cystic lung lesion presented for repeated episodes of respiratory distress, especially during feeding, with a prior bronchoscopy suggestive of tracheomalacia. He demonstrated appropriate weight gain and was meeting developmental milestones. On physical examination, the lungs were rhonchorous to auscultation, and there was evidence of bilateral subcostal retractions and supra-sternal retractions after eating.

**Fig. 1:**
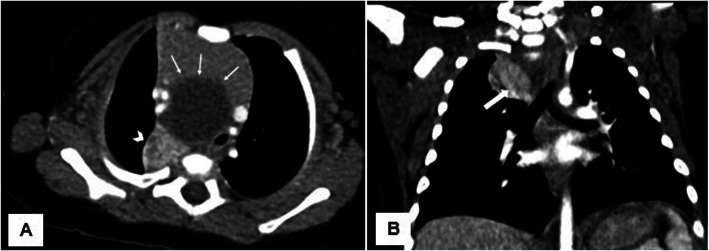
Axial (**a**) contrast-enhanced CT of the chest of a 3-month-old male infant showing a median cystic mass (arrows) causing tracheal deviation and compression, as well as a mass effect on the more anterior thymus, was pathologically demonstrated to be a bronchogenic cyst. A posterior right upper lobe hyperdense consolidation (arrowhead) with anomalous venous drainage to the distal SVC (block arrow), consistent with pulmonary sequestration, is better demonstrated on the coronal image (**b**)

A CT examination of the chest with IV contrast was performed which revealed a 4-cm mediastinal cyst causing tracheal deviation and compression, suspicious for a thymic epithelial cyst versus a bronchogenic cyst. In the right upper lobe, there was an enhancing consolidation with anomalous venous drainage into the distal superior vena cava (SVC), suspicious for pulmonary sequestration (Fig. 1).

**Fig. 2:**
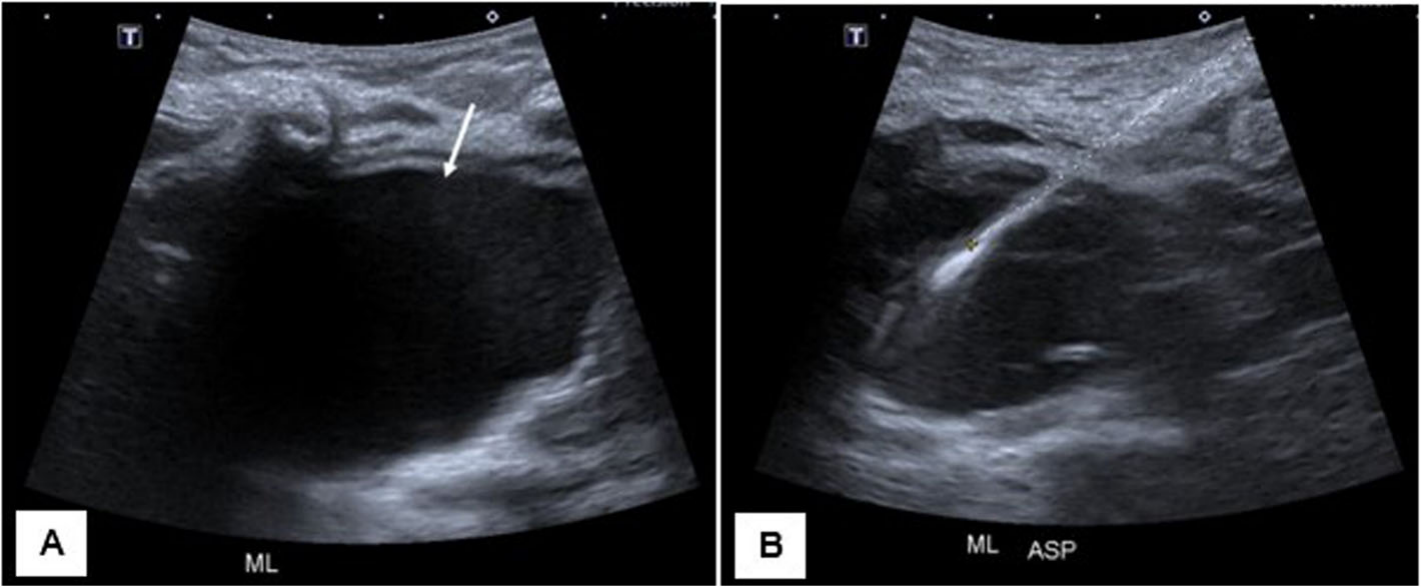
Gray-scale sonographic images of the upper mediastinum in the same patient show a large anechoic cystic structure (arrow) with no internal flow on color Doppler (not shown) (**a**). Approximately 12 ml of clear mucinous fluid was aspirated from the lesion (**b**)

**Fig. 3:**
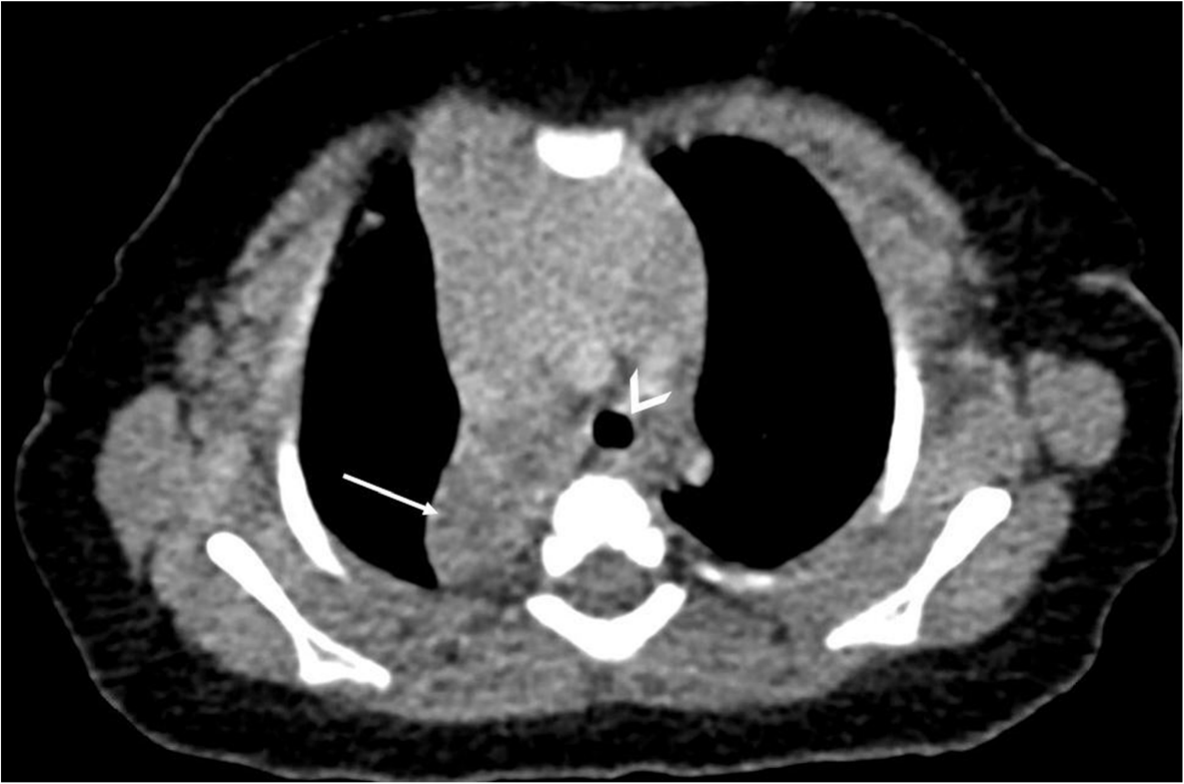
Non-contrast-enhanced axial CT post-aspiration of the mediastinal mass shows significant improvement in the mass effect on the trachea (arrowhead) and thymus. The right upper posterior consolidation concerning pulmonary sequestration (arrow) remains unchanged

The patient was admitted for these findings in the setting of increased work of breathing, and an ultrasound-guided drainage of the cystic mediastinal mass was performed (Fig. 2) with the placement of a drainage catheter. Cytology from the cyst fluid was not concerning for malignancy. Respiratory status improved, and a follow-up CT examination showed near-complete resolution of the cystic mass with significant improvement in mass effect on the adjacent thymus, great vessels, and trachea (Fig. 3).

**Fig. 4:**
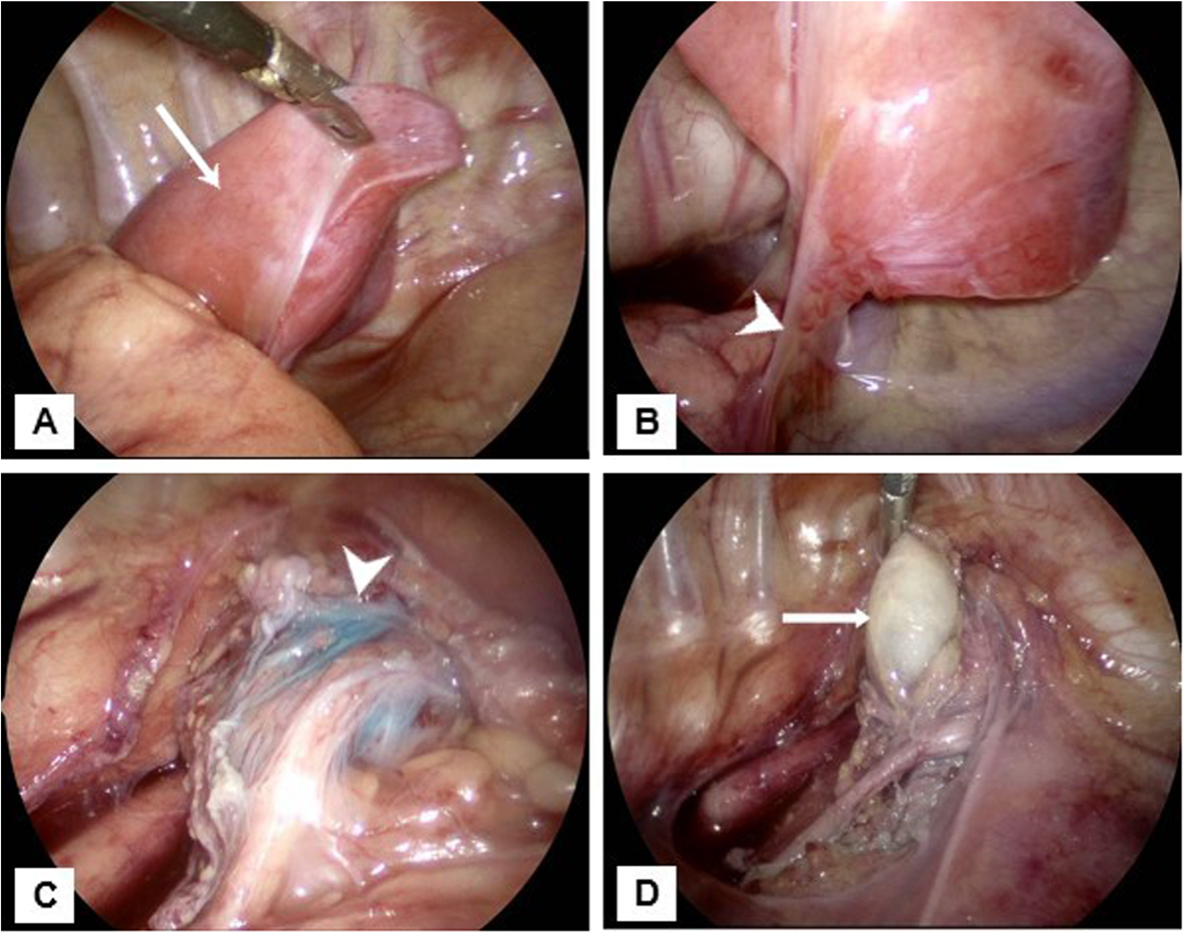
Intraoperative images during thoracoscopy with visualization of an extralobar pulmonary sequestration (arrow) in the right upper thorax (**a**) that is invested in its own pleura. The sequestration appears to be connected to the mediastinum via a stalk (arrowheads in **b** and **c**) that courses towards the SVC/azygos junction. The mediastinal cyst (block arrow) which collapsed post-aspiration (**d**) was also resected

**Fig. 5:**
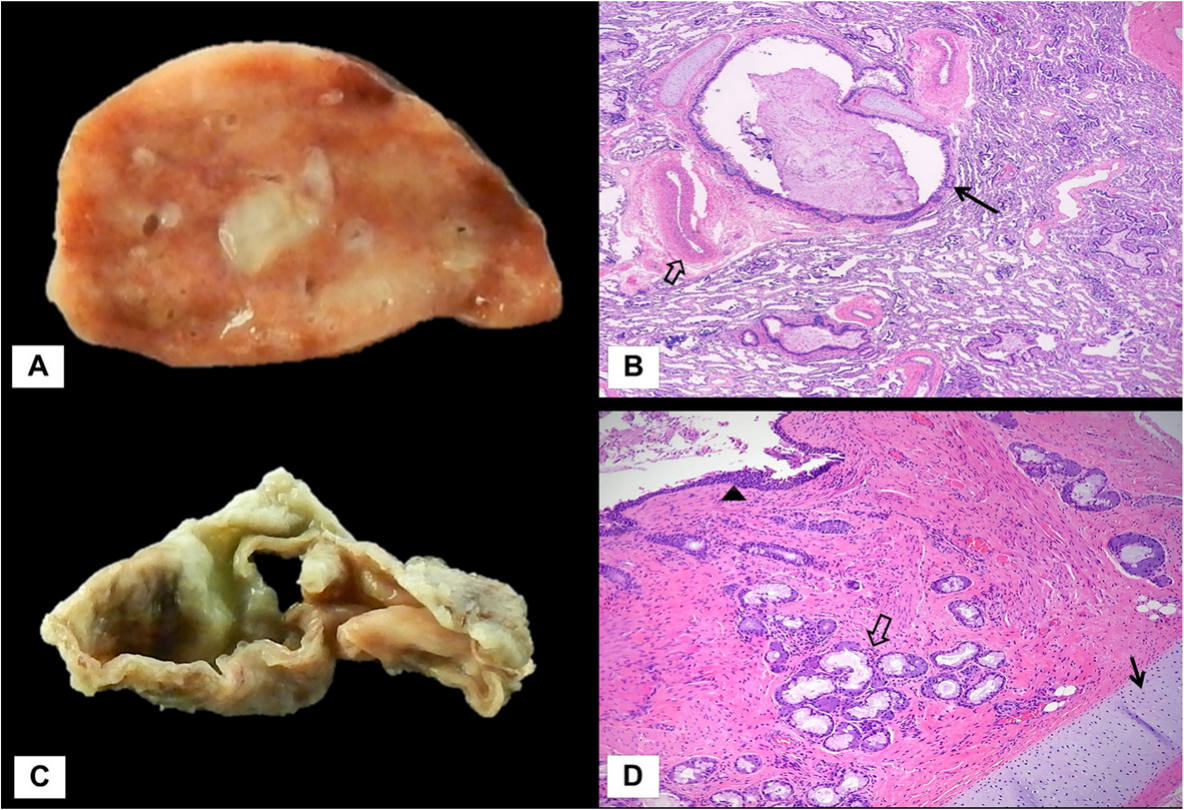
Gross image demonstrating a cross-section of the extralobar pulmonary sequestration, demonstrating focal cystic lung parenchyma (**a**). Microscopic image demonstrating cystic lung parenchyma with extravasated mucin (arrow) and thick-walled vessels (open arrow), × 20 magnification (**b**). Gross image demonstrating a cross-section through the bronchogenic cyst (**c**). Microscopic image demonstrating cyst wall (arrowhead) with bronchial glands (open arrow) and cartilage (arrow), compatible with bronchogenic cyst, × 40 magnification (**d**)

Subsequently, the patient underwent right thoracoscopy and resection of the cystic mediastinal mass and the right upper thoracic mass with a pedicle that extended towards the SVC/azygos junction (Fig. 4). Gross pathology demonstrated a 3.0 × 2.5 × 1.1 cm segment of lung invested by visceral pleura consistent with extralobar pulmonary sequestration. Microscopic examination revealed focal cystic lung parenchyma with extravasated mucin and interspersed thick-walled blood vessels. A second specimen demonstrated a collapsed cystic structure which on histology showed a lining of ciliated columnar respiratory type epithelium and the cyst wall including bronchial glands and cartilage, consistent with a bronchogenic cyst (Fig. 5). The patient was extubated 1 day after the operation and discharged after 2 days.

Follow-up was delayed related to COVID-19 circumstances. The patient is due for a short-interval follow-up CT examination of the chest.

## Discussion

Bronchopulmonary foregut malformations (BPFMs) are a wide-encompassing term referring to several types of anomalies of pulmonary development resulting from abnormal budding of the embryonic foregut and tracheobronchial tree. BPFMs include foregut duplication cysts, pulmonary sequestrations, and congenital pulmonary airway malformations (CPAMs) [2].

Foregut duplication cysts arise from abnormal budding of the embryonic foregut and tracheobronchial tree and can be classified into three groups: bronchogenic, neuroenteric, and enteric cysts. Bronchogenic cysts arise from abnormal budding of the bronchial tree around 4–6 weeks gestation and are lined by secretory respiratory epithelium [3]. Esophageal duplication cysts, a type of enteric cyst, arise from the posterior division of the embryonic foregut at 3–4 weeks gestation and are lined by gastric epithelium [2].

Pulmonary sequestration is defined as the aberrant formation of segmental lung tissue that has no connection with the bronchial tree or pulmonary arteries [4]. It receives systemic arterial blood supply, usually from the thoracic or abdominal aorta. Venous return can either be pulmonic or systemic. There are two types of sequestration: extralobar pulmonary sequestration which has its own distinct pleural covering and complete separation from adjacent lung tissue, and intralobar pulmonary sequestration which is embedded in the normal lung [4, 5]. There is a predilection for the posterior inferior chest, with 90% of extralobar sequestration occurring on the left side. This contrasts with our patient who was found to have extralobar sequestration in the right superior chest.

Embryology could help to explain the occurrence of some of these congenital anomalies simultaneously. Between 3 and 5 weeks of gestation, the tracheobronchial tree is formed by a median bud that forms on the ventral wall of the pharynx and grows caudally to form the right and left lung buds [4, 5]. As these lung buds elongate, lateral ridges form between the lung buds and dorsal foregut to create the tracheoesophageal septum which separates the esophagus from the trachea. The presence of supernumerary lung buds that arise from the primitive foregut caudal to the normal lung buds is considered the most common factor in the development of all forms of BPFMs [3]. Which type of BPFM develops depends on (1) the stage of embryological development when the accessory tissue arises, (2) the direction in which the aberrant pulmonary tissue grows, and (3) the retention or involution of the communication between the accessory lung tissue and the parent viscus [3].

Foregut duplication cysts arise during the same period of embryogenesis as that of the development of the lung buds. While pulmonary sequestrations are already a very rare malformation with an estimated incidence of 0.15–6.4% [6], extralobar sequestration is less common than intralobar sequestration comprising about 25% of all sequestrations. They have a greater prevalence to be associated with other congenital malformations (65%) than the intralobar type, with the most common being diaphragmatic hernia (16%). Other associations include congenital cystic adenomatoid malformation (CCAM), bronchogenic cysts, pectus excavatum, pericardial defects, and enteric duplication cysts [1]. Very few case studies have cited the rare association between extralobar pulmonary sequestration and bronchogenic cyst [7] or complex bronchopulmonary foregut malformations of the mixed bronchogenic and esophageal type [2, 8]. Most of these lesions occurred in the left hemithorax [2, 7].

Surgical resection is the treatment of choice where extralobar sequestrations can usually be removed without harm to normal lung tissue because it is invested in its own pleura. Surgical excision was also considered the treatment of choice for bronchogenic cysts, especially symptomatic lesions, although more recently, less invasive methods are advocated including videothoracoscopy, mediastinoscopy and the percutaneous approach [9].

## Conclusions

Bronchopulmonary foregut malformations are rare, with the most common malformations of the lower respiratory tract being CPAMs and bronchopulmonary sequestration [10]. The combination of two malformations concurrently is even more rare. Very few cases have been reported in the literature that describes extralobar pulmonary sequestration and bronchogenic cyst, such as the case with our patient and only one case report we found demonstrated a complex malformation in the right inferior hemithorax with pulmonary sequestration and esophageal lung [8]. We did not find any reports of the occurrence of a *right-sided* extralobar sequestration (65% are left-sided) with bronchogenic cyst. Surgery remains the treatment of choice in symptomatic cases, which was seen with our patient who developed respiratory distress likely related to the mass effect of the mediastinal bronchogenic cyst on the adjacent airway.

## Data Availability

Not applicable.
